# A data-driven pipeline to extract potential adverse drug reactions through prescription, procedures and medical diagnoses analysis: application to a cohort study of 2,010 patients taking hydroxychloroquine with an 11-year follow-up

**DOI:** 10.1186/s12874-022-01628-3

**Published:** 2022-06-08

**Authors:** P. Sabatier, M. Wack, J. Pouchot, N. Danchin, AS. Jannot

**Affiliations:** 1 Inria, HeKA, PariSantéCampus, 10 Rue d’Oradour-sur-Glane, 75015 Paris, France; 2 Inserm, Centre de Recherche des Cordeliers, Sorbonne Université, Université de Paris Cité, 75006 Paris, France; 3 AP-HP: Medical Informatics Department, Georges Pompidou European Hospital, 20 Rue Leblanc, 75015 Paris, France; 4 AP-HP: Department of Cardiology, Georges Pompidou European Hospital, 75015 Paris, France; 5 AP-HP: Department of Internal Medicine, Georges Pompidou European Hospital, 75015 Paris, France

**Keywords:** Hydroxychloroquine, Adverse drug reactions, Medico-administrative databases, Massive data

## Abstract

**Context:**

Real-life data consist of exhaustive data which are not subject to selection bias. These data enable to study drug-safety profiles but are underused because of their temporality, necessitating complex models (i.e., safety depends on the dose, timing, and duration of treatment). We aimed to create a data-driven pipeline strategy that manages the complex temporality of real-life data to highlight the safety profile of a given drug.

**Methods:**

We proposed to apply the weighted cumulative exposure (WCE) statistical model to all health events occurring after a drug introduction (in this paper HCQ) and performed bootstrap to select relevant diagnoses, drugs and interventions which could reflect an adverse drug reactions (ADRs). We applied this data-driven pipeline on a French national medico-administrative database to extract the safety profile of hydroxychloroquine (HCQ) from a cohort of 2,010 patients.

**Results:**

The proposed method selected eight drugs (metopimazine, anethole trithione, tropicamide, alendronic acid & colecalciferol, hydrocortisone, chlormadinone, valsartan and tixocortol), twelve procedures (six ophthalmic procedures, two dental procedures, two skin lesions procedures and osteodensitometry procedure) and two medical diagnoses (systemic lupus erythematous, unspecified and discoid lupus erythematous) to be significantly associated with HCQ exposure.

**Conclusion:**

We provide a method extracting the broad spectrum of diagnoses, drugs and interventions associated to any given drug, potentially highlighting ADRs. Applied to hydroxychloroquine, this method extracted among others already known ADRs.

**Supplementary Information:**

The online version contains supplementary material available at 10.1186/s12874-022-01628-3.

## Highlights

• The challenge of drug-safety signal detection methods is to handle four types of difficulties:

*○* The data source, the study of long-term adverse drug reactions or effects not suspected by healthcare professionals, requires the use of a real-life data source.

*○* The consideration of a broad spectrum of potential adverse drug reactions (ADRs), and not only candidate ADRs.

*○* The temporal impact (meaning that safety depends on the dose, date and duration of treatment).

*○* The difference between true ADRs and disease natural course.

• We aimed to create a data-driven pipeline strategy, without any assumption of any ADRs, which take into account the complex temporality of real-life data to provide the safety profile of a given drug.

• Our pipeline used three sources of real-life data to establish a safety profile of a given drug: drug prescriptions, procedures and medical diagnoses.

• We successfully applied our data-driven pipeline strategy to hydroxychloroquine (HCQ). Our pipeline enabled us to find diagnoses, drugs and interventions related to HCQ and which could reflect an ADR due to HCQ or the disease itself.

• This data-driven pipeline strategy may be of interest to other experts involved in the pharmacovigilance discipline.

**Supplementary Information:**

The online version contains supplementary material available at 10.1186/s12874-022-01628-3.

## Introduction

Adverse drug reactions (ADRs) have been attributed to causing over 770,000 injuries and 100,000 deaths and $76.6 billion in annual costs in the US [1], responsible for 143,915 hospitalizations and are estimated to 10,000 deaths in France [2]. The rapid detection of ADRs has become a crucial public health issue. Currently, drug safety is monitored by the pharmacovigilance system, which uses spontaneous reporting systems (SRSs) to detect, collect, and analyze ADRs. However, SRSs suffer from underreporting. Indeed, less than 10% of serious ADRs are reported [3, 4]. Moreover, this system is subject to biases due to selective reporting (most of the reported cases are considered as suspected ADRs) or by a reporting bias for newly marketed medicines.

The increasing availability of electronic medical records (EMR) offers major opportunities to investigate a wide spectrum of ADRs and detect drug safety signals closer to real use and time, as EMR databases record information for large populations over long follow-up periods [5]. These databases, such as electronic medical records and administrative claims databases, have been mostly used to confirm or disprove potential signals flagged by SRSs. A number of data-mining techniques have been specifically developed for the automatic detection of drug-safety signals using either SRS or EMR databases [6–12]. Over the last decade, several international initiatives have been developed; the Mini-Sentinel and OMOP (Observational Medical Outcomes Partnership) in the United States and the PROTECT (Pharmaco-epidemiological Research on Outcomes of Therapeutics by a European Consortium) and EU-ADR (Exploring and Understanding Adverse Drug Reactions) in Europe.

The challenge of drug-safety signal detection methods is to handle four types of difficulties. The first difficulty is the data source. The study of long-term adverse drug reactions or effects not suspected by healthcare professionals requires the use of a real-life data source, such as EMR or claims databases, which do not suffer from the known bias of underreporting and reporting selection [13, 14]. The second difficulty is the consideration of a broad spectrum of potential ADRs, and not only candidate ADRs [15], to be able to highlight new signals. The third difficulty is to precisely take into account the temporal aspect. Time is important, because the type of adverse reactions caused by the medication under study may differ according to the duration of the medication prescriptions. Certain adverse effects may occur soon after the start of the medication under study, whereas others may require a prolonged period of administration to become manifest [16]. Safety depends on the dose, timing, and duration of the treatment. The last difficulty concerns distinguishing true ADRs from the natural course of the disease. Indeed, the natural course of the disease for which the prescribed drug is indicated may be associated with many other medical diagnoses, which may be indistinguishable from ADRs.

In this study, we developed a data-driven pipeline based on a cumulative exposure test to highlight relevant diagnoses, drugs and interventions which could reflect an ADR of a given drug. We used the French national medication reimbursement database to apply this pipeline to HCQ, an old drug with widely known side effects.

### Description of the WCE-data-driven pipeline

The WCE-data-driven pipeline aims to overcome the four aforementioned difficulties as follows:

− Data source: Our method is dedicated to exhaustive real-life data encompassing the whole medical pathway of each patient after drug intake and all along its use.
− Agnostic approach: all elements of the medical pathway are considered with no expert preselection
− Temporality assessment: We modeled the increase in risk related to cumulative dose using splines in Cox proportional hazards models [17, 18]. Dose during a month is defined as having had at least one reimbursement for the drug of interest during this month. This model is called weighted cumulative exposure (WCE). It allows the representation of complex cumulative effects of dose, timing, and duration of interest drug [19].
− ADRs denoising: We introduced a covariate representing disease severity built from expert knowledge to automatically pinpoint drugs given for the condition for which the exposure drug has been prescribed.

We will first introduce this WCE-data-driven pipeline, followed by a presentation of a comparative method to our WCE-data-driven pipeline. Finally, we present a use case of this pipeline and compared it with a more classical approach.

### WCE-data-driven pipeline

The WCE-data-driven pipeline applies to a case-only cohort, i.e. all patients in the cohort were exposed at least once during the study period to the drug of interest.

**Fig. 1:**
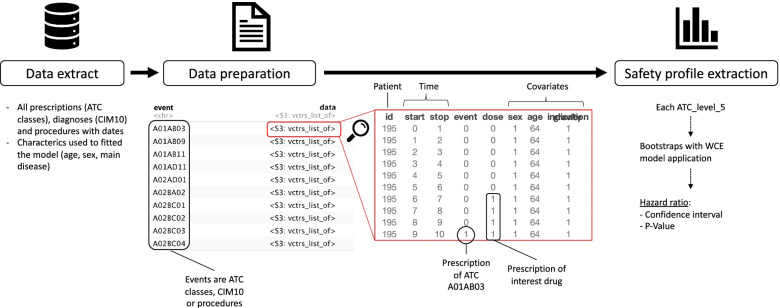
Flow chart of WCE-data-driven pipeline

The WCE-data-driven pipeline is divided into three steps: data extraction, data preparation, and safety-profile extraction (Fig. 1).

- i. **Data extraction**: All drug prescriptions for the drug under study are extracted, along with all health events (treatment initiation date of all drugs, procedure dates, medical diagnoses dates), and patient characteristics, such as age, sex.
- ii. **Data preparation**: We built a data frame corresponding to the WCE package [20] data format for each possible type of event occurring after the first prescription of the drug under study. In each data frame, each row corresponds to a given time period, such as day, week, or month (to be defined). Id identifies the patients. Start and Stop identify the beginning and end of each interval (Stop in row n = Start in row n+1). The intervals are closed on the right. For each patient, the first start (start = 0) is the start date of the study period. The last start is the date the patient has the event or the date of the last patient follow-up. Event is a binary indicator for the event of interest, which has a value of 1 if the event occurred in the interval specified by Start and Stop. For a given subject, event = 1 can only occur in the last interval of his or her follow-up. The following columns represent patient covariates.
- iii. **Safety-profile extraction**: The weighted cumulative exposure (WCE) model is applied to estimate the cumulative effect of duration, dose, and prescription date for the drug of interest [19]. The WCE statistical model approach estimates the effect of past exposure using a weighted sum of all previous instances of exposure, with weights depending on the time elapsed since exposure and the dose [18, 21]. This makes it possible to account for the fact that the type of adverse reactions caused by the medication under study may depend on the duration of the medication prescription (certain adverse effects may occur soon after the start of the medication under study, whereas others may require a prolonged period of administration to become manifest). WCE statistical model allow taking into account for patient’s covariates as relying on Cox time-dependent in which initial covariates might be introduced.

**Fig. 2:**
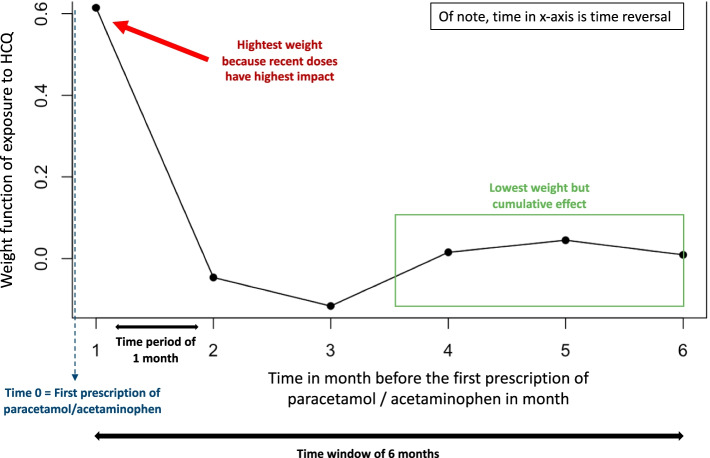
For example, weight function estimated by the WCE model for the risk of paracetamol prescription (event) after HCQ exposure (interest drug). Each point on the curve represents the estimated value of the exposure weight function of the interest drug (HCQ) for each time (here month) of the time window (here six months). The time zero on the x-axis corresponds to the event (the first prescription of paracetamol/acetaminophen)

The WCE statistical model works in two steps. First, it estimates the weighting function that assigns weights to past exposures (e.g. dose or intensity of exposure) as a function of time since exposure, using cubic regression splines (Fig. 2).

Then, a Cox proportional hazards model is fitted to compare a group exposed to the drug of interest (HCQ in this example) during the time window to a group not exposed during the same period [20]*.*

To select health events significantly associated with exposure, confidence intervals for each hazard ratio are needed. Initially, WCE model was not designed to test association with an ADR but to assess risk function of a known association, therefore we developed an association test for WCE using bootstrapping to estimate both the confidence intervals and P-values of the exposure and of the initial covariate’s coefficient (number of repeats to be defined). We constructed bootstrap replicates of the data, each of which was randomly sampled with a replacement. Confidence intervals were estimated using the percentile method:

$$[{\theta }_{lower limit},{\theta }_{upper limit}]=[{\theta }_{j}^{*}, {\theta }_{k}^{*}]$$

*Equation 1: Confidence intervals, where *${\theta }_{j}^{*}$* denotes the jth quantile (lower limit) and *${\theta }_{k}^{*}$* denotes the kth quantile (upper limit), j* = *[α/2* × *B], k* = *[(1-α/2)* × *B], B random bootstrap samples.*

All analyses were performed using R (version 4.0.3) and WCE package (version 1.0.2).

### WCE competitor

As the above pipeline is not specific to the method used to assess association between the studied drug and each diagnoses, drugs and interventions which could reflect an ADR, we also implemented case-crossover [22, 23].

We chose a case-crossover design for WCE competitor because it is a method adapted to a case-only cohort and this design is widely used in pharmacoepidemiology because it is not sensitive to unmeasured, time-invariant confounding factors.

**Fig. 3:**
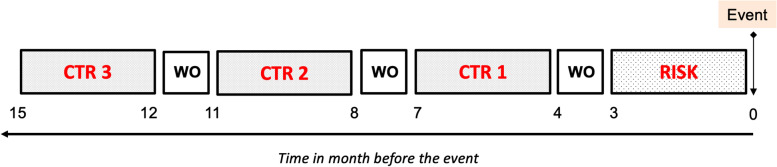
Diagram of case-crossover. Schematic representation of a case–crossover analysis. The case-defining event is the prescription of a level 5 ATC class other than HCQ and the prescription is the HCQ drug. Three control periods are selected (shown as CTR) and one risk period (shown as RISK) for each individual. The duration of the periods (risk and control) is set to three months in this example. The washout period (shown as WO) has a standard duration of one month

Case-crossover requires defining risk periods and a wash-out period. Four periods were defined for each individual, separated by a washout period: one risk period and three control periods. Each period had the same duration (Fig. 3). We performed a sensitivity study by varying the duration of these periods (risk and control) of three, six and nine months, with a washout period of one month.

### Use case

**Fig. 4:**
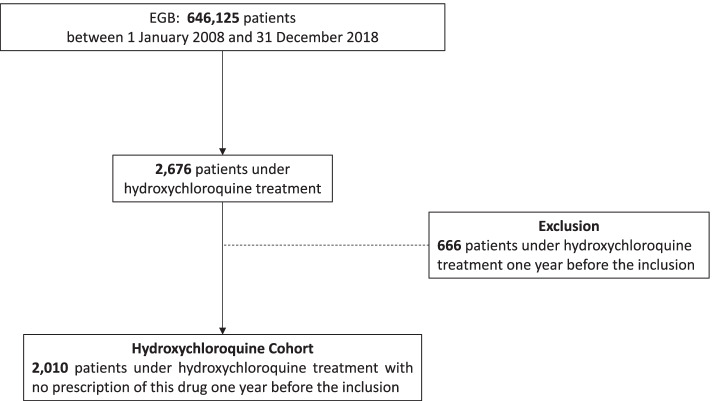
Population flowchart of hydroxychloroquine cohort

In the present study, we applied our data-driven pipeline to patients newly exposed to HCQ (considered in WCE as the exposure variable). The cohort of patients receiving HCQ was extracted from the EGB (Echantillon Généraliste de Bénéficiaires), a permanent 1/97 representative sample of the Système National d’Informations Inter-régimes de l’Assurance Maladie (SNIIRAM), which includes the data for 66 million people. The EGB includes data for approximately 780,000 people [24], consisting of de-identified data on demographic characteristics (sex, year of birth, date of death), long-term diseases (ALD), and reimbursed acts (visits, medical procedures, laboratory tests, dispensed drugs, medical devices) [24–26]. We extracted the data of all patients with at least one reimbursement for HCQ. We only included patients who did not receive HCQ during the previous year to select only newly exposed patients (Fig. 4). For these patients, we extracted the following data: age, sex, date of all HCQ deliverances, first date of prescription for all other drugs, date of procedures and medical diagnoses, date of “chronic disease certification status”, which is a marker of disease severity in the French health system.

All the parameters that were used in our case study are presented in Table 1.

**Table 1:** Parameter set-up

Parameter	Set-up in our study	Explanation
Drug of interest	Hydroxychloroquine	drug for which we want to know all co-prescriptions after exposure
Study period	01/01/2008 to 12/31/2018	study start and stop
Time period	month	time interval between start and stop
Time window	24 months	potential exposure period before the event
Covariates	age, sex, and severity	covariate to fit the model
nknots	1	number of interior knots for the cubic spline to estimate the risk function
Bootstraps	1,000	repeat on sample of the WCE model application
Number of ATC classes	1,081	ATC classes level 5
Number of medical diagnoses	4,200	code CIM10
Number of procedures	3,075	we used “classification commune des actes médicaux”, a classification from the French National Insurance

We assume that a more severe disease leads to a higher use of some drugs, diagnoses or interventions. Therefore, severity of the disease might be a confusion factor for the relationship between the drug of interest and some other drugs, diagnoses and interventions as it is associated with the cumulative dose of the drug of interest and to other drugs, diagnoses and interventions related to disease severity. Therefore, when it is not included in the model, all drugs, diagnoses and interventions linked to disease severity will be highlighted using our model, resulting in many positive results that are not likely to be ADRs. To limit these signals, we have created a disease severity variable to improve the specificity of the detected signals. The disease severity variable is designed manually from expert knowledge and depends only on the drug of interest. In our HCQ use case, the disease severity variable was defined as having "chronic disease certification status" for lupus or rheumatoid arthritis. The creation of a disease severity variable is a non-mandatory step and is added in the model like other confounding factors such as age and sex.

The EGB database contains only anonymized data and its access is legally authorized without having to receive authorization from the national data protection agency (CNIL). The study protocol was submitted to the appropriate INSERM and CNAMTS entities, as legally required. In addition, the study was approved by our Institutional Review Board (CER-APHP CENTRE, IRB n°20,180,603).

For this use case, we considered age, sex and disease severity as covariates. The significance level chosen was 5% because this was an exploratory use case, therefore we did not correct for multiple testing.

Significant associations were then compared with recorded side effects available in drug databases of Food Drug Administration (FDA) organization (source: https://www.accessdata.fda.gov/scripts/cder/daf/index.cfm?event=overview.process&ApplNo=009768).

## Results

### Population description

The HCQ cohort contains 2,010 patients (n_women_ = 1,577, 78%), with 1,081 different ATC classes, 4,200 different diagnostics and 3,075 different procedures. The average age at the time of the first prescription of HCQ was 54.8 (sd = 16.2). Within the cohort, 1,045 (52%) patients declared their disease to National Insurance to get full-price reimbursement for all treatments related to their chronic disease. Among them, 12% (*n* = 240) each had vasculitis, systemic lupus erythematous, or systemic scleroderma, 11% (*n* = 214) rheumatoid arthritis, and 6% (*n* = 122) each malignant neoplasm or malignant disease of the lymphatic or hematopoietic tissue (Table 2). There was an association between age and HCQ exposure (*P* < 0.001 by linear regression analysis), as well as between sex and HCQ (*P* < 0.001 by the Wilcoxon test). There was also a strong association between HCQ exposure and severity (*P* < 0.001 by the Wilcoxon test).

**Table 2:** Population characteristics

Description	Hydroxychloroquine Cohort
**n (patients)**	2,010
number of women (%)	1,577 (78%)
mean age (IQR)	54.8 (23)
number of severe patients (%)	701 (35%)
mean exposure, in months (sd)	9.5 (16.1)
vasculitis, systemic lupus erythematous, systemic scleroderma	240 (12%)
rheumatoid arthritis	214 (11%)
malignant neoplasm, malignant disease of the lymphatic or hematopoietic tissue	122 (6%)

### Application of the WCE-data-driven pipeline

**Fig. 5:**
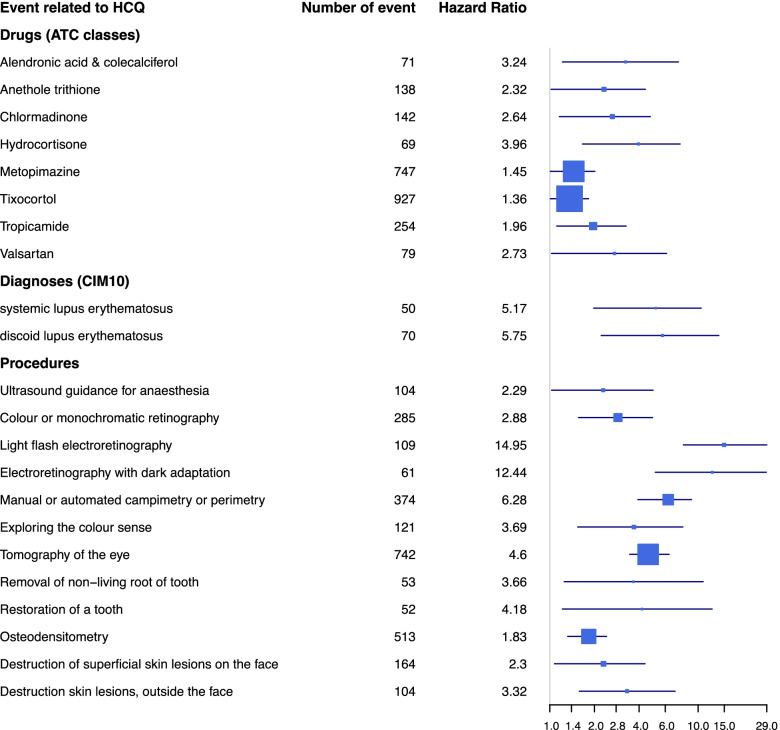
Forest plot of the effect of hydroxychloroquine on ATC classes, medical diagnoses and procedures with the WCE model

The WCE-data-driven pipeline enabled the identification of eight ATC classes, twelve procedures and two medical diagnoses associated with the prescription of HCQ, of which seven were borderline significant at 5% significance level. The highest risk ratios ATC classes were obtained for hydrocortisone (HR = 3.96 [1.66–7.55]), alendronic acid/cholecalciferol (HR = 3.24 [1.22–7.36]), valsartan (HR = 2.73 [1.03–6.13]), and chlormadinone (HR = 2.65 [1.16–4.76]). The highest risk ratios of diagnoses were obtained for systemic lupus erythematous, unspecified (HR = 5.75 [2.22–13.77]) and discoid lupus erythematous (HR = 5.178 [1.98–10.47]). The highest risk ratios procedures were obtained for light flash electroretinography with measurement of response amplitudes and latencies (HR = 14.95 [7.95–28.99]), electroretinography with dark adaptation (HR = 12.44 [5.14–28.75]), manual or automated campimetry or perimetry, with specific threshold measurement programs (HR = 6.28 [3.93–9.03]), unilateral or bilateral optical coherence tomography of the eye (HR = 4.60 [3.46–6.37]). (Fig. 5 & Additional file 1 Appendix 1). In additional file 1 appendix 2, we have represented the risk functions for light flash electroretinography procedure and electroretinography with dark adaptation procedure.

### Application of the case-crossover-data-driven pipeline

With risk periods set up at three months, the case-crossover competitor method identified nine ATC classes, the highest risk ratios were obtained for diclofenac (HR = 1.40 [1.14–1.72]), prednisone (HR = 1.31 [1.07–1.60]), racecadotril (HR = 1.26 [1.03–1.53]) and cholecalciferol (HR = 1.26 [1.03–1.54]); zero medical diagnoses and twelve procedures, the highest risk ratios were obtained for unilateral or bilateral optical coherence tomography of the eye (HR = 1.74 [1.44–2.09]), manual or automated campimetry or perimetry, with specific threshold measurement programs (HR = 1.65 [1.38–1.97]), fundus examination by biomicroscopy with contact lens (HR = 1.49 [1.23–1.80]) and supplement for carrying out a digitised X-ray examination (HR = 1.46 [1.17–1.82]). Results for risk periods set up at three months, six months and nine months are presented in additional file 1 appendix 3.

In total, no ATC class, no medical diagnosis and four procedures were common between WCE and case-crossover methods: unilateral or bilateral optical coherence tomography of the eye, manual or automated campimetry or perimetry, with specific threshold measurement programs, exploring the color sense by matching and light flash electroretinography, with measurement of response amplitudes and latencies.

### Comparison with known side effects

Related association signals were found for all very common and common known side effects (Table 3), except headache for which indicated drugs such as acetaminophen are over-the-counter treatments and lability disorders and anorexia for which there is no indicated treatment for acute events. No related association signals were found for uncommon side effects and side effects for which prevalence is not known, except for maculopathies, for which checking procedure was found to be associated (but not treatment for the side effect himself).

**Table 3:** HCQ adverse drug reactions know

Localization	Adverse effect	Frequency	Related association signals
**Gastrointestinal disorders**	Abdominal pain, nausea	*Very common*	metopimazine
	Diarrhea, vomiting	*Common*	metopimazine
**Eye disorders**	Blurring of vision due to a disturbance of accommodation	*Common*	eyes procedures
	Retinopathy with changes in pigmentation and visual field defects	*Uncommon*	eyes procedures
	Cases of maculopathies and macular degeneration have been reported	*Not known*	eyes procedures
**Cardiac disorders**	Cardiomyopathy which may result in cardiac failure and in some cases a fatal outcome	*Not known*	No signal
**Ear and labyrinth disorders**	Vertigo, tinnitus	*Uncommon*	No signal
	Hearing loss	*Not known*	No signal
**Nervous system disorders**	Headache	*Common*	No signal
	Dizziness	*Uncommon*	No signal
	Convulsions have been reported with this class of drugs	*Not known*	No signal
**Psychiatric disorders**	Affect lability	*Common*	No signal
	Nervousness	*Uncommon*	No signal
	Psychosis	*Not known*	No signal
**Immune system disorders**	Urticaria, angioedema, bronchospasm	*Not known*	No signal
**Blood and Lymphatic system disorders**	Bone-marrow depression, anemia, aplastic anemia, agranulocytosis, leucopenia and thrombocytopenia	*Not known*	No signal
**Hepatobiliary disorders**	Abnormal liver function tests	*Uncommon*	No signal
	Fulminant hepatic failure	*Not known*	No signal
**Skin and subcutaneous tissue disorders**	Skin rash, pruritus	*Common*	No signal
	Pigmentation disorders in skin and mucous membranes, bleaching of hair, alopecia	*Uncommon*	No signal
	Bullous eruptions including erythema multiforme, Stevens-Johnson syndrome and toxic epidermal necrolysis, Drug Rash with Eosinophilia and Systemic Symptoms, photosensitivity, exfoliative dermatitis, acute generalised exanthematous pustulosis	*Not known*	No signal
**Musculoskeletal and connective tissue disorders**	Skeletal muscle myopathy or neuromyopathy leading to progressive weakness and atrophy of proximal muscle groups	*Not known*	No signal
**Metabolism and nutrition disorders**	Anorexia	*Common*	No signal
	Hypoglycemia	*Unknown*	No signal

Very common (≥ 1/10), Common (≥ 1/100 to < 1/10), Uncommon (≥ 1/1,000 to < 1/100), Rare (≥ 1/10,000 to < 1/1,000), Very rare (< 1/10,000), not known (cannot be estimated from the available data)

## Discussion

We report a new data-driven pipeline able to pinpoint diagnoses, drugs and interventions associated to any given drug and makes it possible to highlight unexpected associations that could represent ADRs. The proposed WCE-pipeline compared to other methods allows the use of the whole prescription trajectories of the drug under study, and therefore allows highlighting both acute and cumulative effects of the drug under study, and to take into account dose–effect relationship, which is essential to infer causal association. This is not the case for methods commonly used to detect ADRs using administrative database such as case-crossover or prescription sequence symmetry analysis (PSSA) [27], making it more powerful to analyze the whole ADRs spectrum. Moreover, our data-driven pipeline is based on a self-controlled method, thus avoiding the usual biases of methods such as case–control [28] or propensity score [29] based approaches.

A major strength of the proposed pipeline is the ability to use a medico-administrative claims, which are not biased towards voluntary declaration, enabling to detect unexpected side effect [30]. Compared to the use of hypothesis-based pharmacovigilance strategy, this pipeline required an additional interpretation step to distinguish in associated signals, those unexpected from both known side effects and signals related to medical condition(s) for which the treatment is prescribed. Thus, this pipeline, applied to HCQ on a cohort of 2,010 patients, allowed us to identify most common and very common side effects, but also associations related to the medical conditions for which HCQ is prescribed: anethole trithione, a bile secretion-stimulating drug restores salivation and relieves the discomfort of dry mouth in Sjögren's syndrome, often associated with lupus [31]; hydrocortisone, a glucocorticoid, is often used for withdrawal from more potent corticosteroids in the treatment of lupus or rheumatoid arthritis; chlormadinone, a progestin macro-pill is recommended as a contraceptive method for women with lupus [32, 33]; alendronic acid/cholecalciferol and osteodensitometry procedure are also a co-prescription of HCQ for optimal management of patients with lupus or rheumatoid arthritis, mostly post-menopausal women, by internists or rheumatologists; this is the same for tooth restoration procedures, as osteoporosis treatment requires a dental check to avoid jawl osteonecrosis and also for skin lesions removal as dermatological consult are part from optimal care in lupus; tixocortol, a glucocorticoid, is a nasal spray treating allergic rhinitis, a common comorbidity in patients suffering from lupus [34]; valsartan, specific angiotensin II (Ang II) receptor antagonist, is used to treat hypertension, a comorbidity of post-menopausal women [35, 36].

Another advantage of our method is that it is a case-only design, therefore it does not imply to choose an unexposed group which is always a very tricky issue, and it does not make the assumption like intermittency of drug use or acute event.

There are multiple reasons that can cause an adverse drug reaction, such as metabolic genetic polymorphisms, e.g. in Asian populations drug metabolism is different than in Caucasian population; gender; age; nutrition; drug interactions. Our model can take additional covariates to adjust for, as we did with gender and age. It can also incorporate interaction terms and therefore integrate drug interaction.

In our use case, we define the dose as cumulative months of exposure to the drug of interest (HCQ) as our data come from a claim database.

This pipeline was however not able to highlight side effects for which no treatment is given neither hospitalization nor procedure is required, but which results in stopping the treatment. No unexpected signal was highlighted using our pipeline. Another limitation of this method is that it is not corrected on multiple tests because our approach is exploratory. Some associations may be due to type-1 error.

However, our use case presents a limitation due to the limited available sample size, resulting in the inability to detect rare side effects. But our pipeline could be applied to larger cohorts when available. Moreover, our pipeline only can be applied when the temporal pattern of the drug under study is available, which is often the case in medico-administrative databases as it collects all drug reimbursements along with their date, while pharmacovigilance databases rarely collect such an information. Our model can work on claim database or cohort with patient prescription, but is not adapted to pharmacovigilance databases like EudraVigilance or FDA Adverse Event Reporting System (FAERS). A limitation of our pipeline is that it uses data from claim databases such as diagnoses or interventions that are intended for reimbursement and does not represent an exhaustive description of patients.

In conclusion, we present a data-driven pipeline that provides a broad picture of diagnoses, drugs and interventions which could reflect an ADR of a given drug. Applied to HCQ, our pipeline allowed us to highlight drugs, procedures and diagnoses associated to HCQ prescription among them most frequent HCQ side effect related events were identified. This is a promising method because true ADRs, co-prescriptions and medical procedures related to the medical condition are found. It would be useful as a complement to traditional pharmacovigilance methods.

## Ethical Approval and Consent to participate

We confirm that this study was approved by an Institutional Review Board IRB (registration: IRB00011928, IRB full name: Comité d'éthique de la recherche CERAPHP Centre IRB #1, study reference: 20,180,603) and has been declared to INSERM (Institut National de la Santé et de la Recherche Médicale, https://www.inserm.fr/). The data from this study are extracted from the EGB (Echantillon Généraliste de Bénéficiaires), a permanent 1/97 representative sample of the National Health Data System (Système National de Données de Santé, SNDS). The information provided to individuals in EGB on the possible reuse of their data and the procedures for exercising their rights comply with the legislative and regulatory provisions applicable to the processing of personal data in the SNDS. According to French regulation, individuals in SNDS database are informed of the reuse of their data for research and can object to this reuse as defined by Articles 92 to 95 of Decree No. 2005–1309 of 20 October 2005 (https://www.legifrance.gouv.fr/loda/article_lc/LEGIARTI000037300884/). As required from French regulation, EGB data can be reused for research projects from authorized persons (ASJ and PS are authorized persons) once the research project is declared to their institution (INSERM).

## Consent for publication

Not applicable.

## Conflict of interest

All authors declare that they have no competing interests in this work.

## Supplementary Information

**Additional file 1:** **Appendix 1.** ATC Class, medical diagnoses and procedures associated withhydroxychloroquine prescription in the WCE model. **Appendix 2:** Risk function of two highestrisk ratios. **Appendix 3:** ATC Class and procedures associated withhydroxychloroquine prescription in the SCCO model. 

## Data Availability

The study was approved by the French data protection agency (Commission Nationale de l’Informatique et des Libertés), but the data access permission policy prohibits making the data set publicly available.
