# Supplementary material for: A data-driven pipeline to extract potential adverse drug reactions through prescription, procedures and medical diagnoses analysis: application to a cohort study of 2,010 patients taking hydroxychloroquine with an 11-year follow-up
Source: BMC Med Res Methodol. 2022 Jun 8;22:166. doi: 10.1186/s12874-022-01628-3 (PMC9175346; doi:10.1186/s12874-022-01628-3)
Supplement: Supplementary file 1 — Additional file 1: Appendix 1. ATC Class, medical diagnoses and procedures associated withhydroxychloroquine prescription in the WCE model. Appendix 2: Risk function of two highestrisk ratios. Appendix 3: ATC Class and procedures associated withhydroxychloroquine prescription in the SCCO model. [file 12874_2022_1628_MOESM1_ESM.docx]

Additional file 1

Appendix 1. ATC Class, medical diagnoses and procedures associated with hydroxychloroquine prescription in the WCE model

| Class ATC | HR [CI_5%_] |
| --- | --- |
| A04AD05 - METOPIMAZINE | 1.45 [1.01-2.01] |
| A16AX02 - ANETHOLE TRITHIONE | 2.32 [1.02-4.41] |
| S01FA06 - TROPICAMIDE | 1.96 [1.12-3.27] |
| M05BB03 - ALENDRONIC ACID AND COLECALCIFEROL | 3.24 [1.22-7.36] |
| H02AB09 - HYDROCORTISONE | 3.96 [1.66-7.55] |
| G03DB06 - CHLORMADINONE | 2.65 [1.16-4.76] |
| C09CA03 - VALSARTAN | 2.73 [1.03-6.13] |
| R01AD07 - TIXOCORTOL | 1.37 [1.01-1.82] |

| Diagnoses | HR [CI_5%_] |
| --- | --- |
| M329 - LUPUS ERYTHEMATOSUS, UNSPECIFIED | 5.75 [2.22-13.77] |
| L930 - DISCOID LUPUS ERYTHEMATOSUS | 5.178 [1.98-10.47] |

| Procedures | HR [CI_5%_] |
| --- | --- |
| BGQP010 - LIGHT FLASH ELECTRORETINOGRAPHY, WITH MEASUREMENT OF RESPONSE AMPLITUDES AND LATENCIES | 14.95 [7.95-28,99] |
| BGQP012 - ELECTRORETINOGRAPHY WITH DARK ADAPTATION | 12.44 [5.14-28.75] |
| BLQP004 - MANUAL OR AUTOMATED CAMPIMETRY OR PERIMETRY, WITH SPECIFIC THRESHOLD MEASUREMENT PROGRAMS | 6.28 [3.93-9.03] |
| BZQK001- UNILATERAL OR BILATERAL OPTICAL COHERENCE TOMOGRAPHY OF THE EYE | 4.6 [3.46-6.37] |
| HBMD047 - RESTORATION OF A TOOTH IN AN INCISIVOCANINE SECTOR ON 2 ANGLES BY A MATERIAL INSERTED IN PLASTIC PHASE, WITHOUT ROOT ANCHORAGE | 4.19 [1.22-12.42] |
| BLQP007 - EXPLORING THE COLOUR SENSE BY MATCHING | 3.69 [1.54-7.88] |
| HBFD001 - REMOVAL OF NON-LIVING ROOT CANAL CONTENTS FROM A PERMANENT INCISOR OR CANINE TOOTH | 3.66 [1.25-10.8] |
| QZNP004 - DESTRUCTION OF 1 TO 10 SUPERFICIAL SKIN LESIONS BY CHEMICAL AGENT OR BY CONTACT CRYOTHERAPY, OUTSIDE THE FACE | 3.32 [1.58-6.99] |
| BGQP007 - COLOUR OR MONOCHROMATIC RETINOGRAPHY, WITHOUT INJECTION | 2.88 [1.56-4.92] |
| QANP007 - DESTRUCTION OF 1 TO 10 SUPERFICIAL SKIN LESIONS ON THE FACE, BY CHEMICAL AGENT OR CONTACT CRYOTHERAPY | 2.3 [1.08-4.38] |
| AHQJ021- ULTRASOUND GUIDANCE FOR ANAESTHESIA | 2.29 [1.03-4.97] |
| PAQK007 - OSTEODENSITOMETRY | 1.83 [1.32-2.41] |

Appendix 2: Risk function of two highest risk ratios


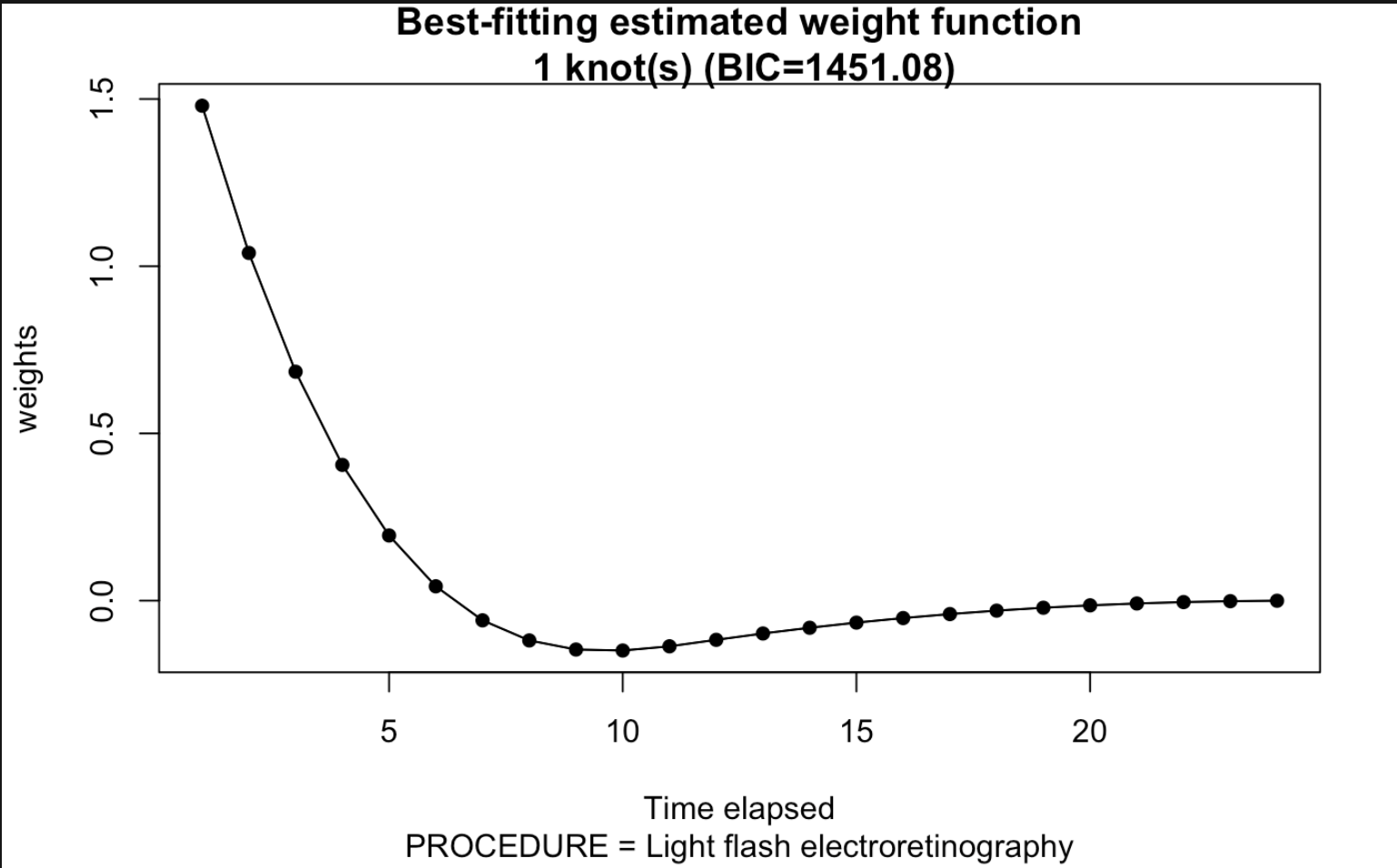


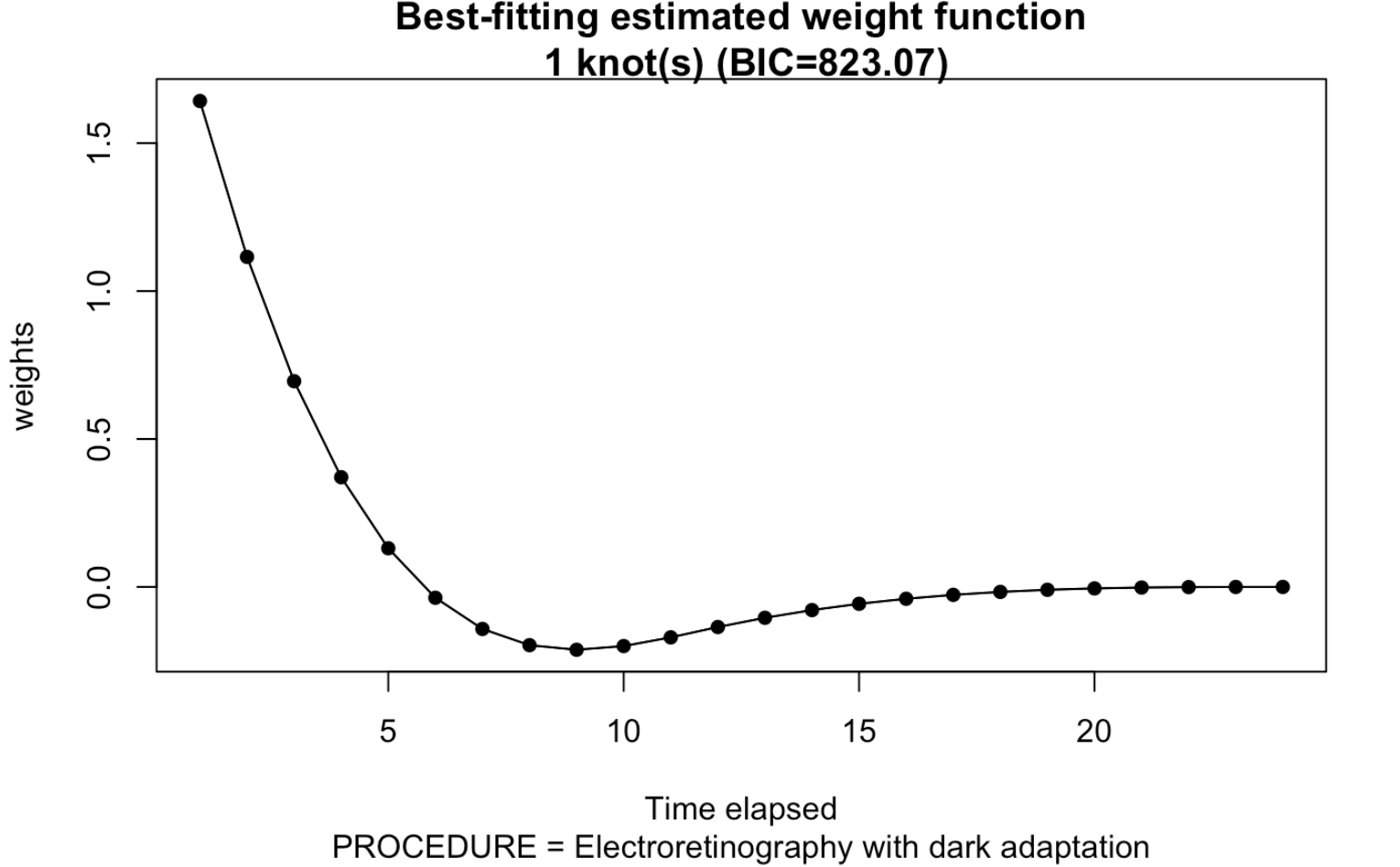


Appendix 3: ATC Class and procedures associated with hydroxychloroquine prescription in the SCCO model

| Class ATC | Period 3 months  HR [CI_5%_] | Period 6 months  HR [CI_5%_] | Period 9 months  HR [CI_5%_] |
| --- | --- | --- | --- |
| M02AA15 - DICLOFENAC | 1.40 [1.14-1.72] | 1.5 [1.27-1.78] | 1.45 [1.24-1.68] |
| H02AB07 - PREDNISONE | 1.31 [1.07-1.60] | 1.61 [1.36-1.9] | 1.67 [1.44-1.94] |
| A07XA04 - RACECADOTRIL | 1.26 [1.03-1.53] | 1.41 [1.21-1.66] | 1.46 [1.27-1.67] |
| A11CC05 - COLECALCIFEROL | 1.26 [1.03-1.54] | 1.5 [1.27-1.77] | 1.64 [1.41-1.9] |
| A02BC01 - OMEPRAZOLE | 1.25 [1.01-1.54] | 1.46 [1.23-1.73] | 1.42 [1.22-1.65] |
| A03AX12 - PHLOROGLUCINOL | 1.24 [1.01-1.53] | 1.45 [1.23-1.71] | 1.55 [1.33-1.79] |
| L04AX03 - METHOTREXATE | 1.24 [1.03-1.49] | 1.5 [1.29-1.74] | 1.55 [1.36-1.77] |
| N05BA12 - ALPRAZOLAM | 1.24 [1.01-1.51] | 1.47 [1.25-1.72] | 1.5 [1.3-1.73] |
| S01XA20 - ARTIFICIAL TEARS AND OTHER INDIFFERENT PREPARATIONS | 1.24 [1.02-1.50] | 1.48 [1.27-1.72] | 1.45 [1.26-1.66] |

| Procedures | Period 3 months  HR [CI_5%_] | Period 6 months  HR [CI_5%_] | Period 9 months  HR [CI_5%_] |
| --- | --- | --- | --- |
| BZQK001- UNILATERAL OR BILATERAL OPTICAL COHERENCE TOMOGRAPHY OF THE EYE | 1.74 [1.44-2.09] | 1.83 [1.58-2.13] | 1.78 [1.55-2.03] |
| BLQP004 - MANUAL OR AUTOMATED CAMPIMETRY OR PERIMETRY, WITH SPECIFIC THRESHOLD MEASUREMENT PROGRAMS | 1.65 [1.38-1.97] | 1.84 [1.6-2.13] | 1.8 [1.58-2.05] |
| BGQP002 - FUNDUS EXAMINATION BY BIOMICROSCOPY WITH CONTACT LENS | 1.49 [1.23-1.80] | 1.59 [1.36-1.85] | 1.58 [1.37-1.81] |
| YYYY030 - SUPPLEMENT FOR CARRYING OUT A DIGITISED X-RAY EXAMINATION | 1.46 [1.17-1.82] | 1.52 [1.28-1.82] | 1.55 [1.33-1.82] |
| DEQP003 - ELECTROCARDIOGRAPHY ON AT LEAST 12 LEADS | 1.41 [1.15-1.73] | 1.45 [1.23-1.7] | 1.46 [1.26-1.69] |
| BLQP007 - EXPLORING THE COLOUR SENSE BY MATCHING | 1.37 [1.13-1.65] | 1.44 [1.24-1.67] | 1.39 [1.22-1.58] |
| DZQM006 - TRANSTHORACIC DOPPLER ULTRASOUND OF THE HEART AND INTRATHORACIC VESSELS | 1.32 [1.08-1.62] | 1.45 [1.23-1.7] | 1.53 [1.33-1.76] |
| QEQM001 - UNILATERAL OR BILATERAL BREAST ULTRASOUND | 1.30 [1.05-1.59] | 1.51 [1.28-1.78] | 1.47 [1.27-1.7] |
| BLQP010 - BINOCULAR VISION TEST | 1.28 [1.05-1.57] | 1.33 [1.12-1.56] | 1.37 [1.18-1.58] |
| BGQP010 - LIGHT FLASH ELECTRORETINOGRAPHY, WITH MEASUREMENT OF RESPONSE AMPLITUDES AND LATENCIES | 1.27 [1.05-1.54] | 1.43 [1.23-1.66] | 1.41 [1.23-1.61] |
| YYYY187 - SUPPLEMENT FOR DIGITAL ARCHIVING OF A RADIOGRAPHIC OR ULTRASOUND EXAMINATION | 1.27 [1.02-1.57] | 1.35 [1.14-1.6] | 1.32 [1.14-1.54] |
| YYYY600 - SUPPLEMENT FOR DIGITAL ARCHIVING OF A MAMMOGRAM OR A SCAN OR REMNOGRAPHIC EXAMINATION | 1.24 [1.01-1.52] | 1.3 [1.1-1.53] | 1.36 [1.18-1.57] |
